# Mumps virus-induced innate immune responses in mouse Sertoli and Leydig cells

**DOI:** 10.1038/srep19507

**Published:** 2016-01-18

**Authors:** Han Wu, Lili Shi, Qing Wang, Lijing Cheng, Xiang Zhao, Qiaoyuan Chen, Qian Jiang, Min Feng, Qihan Li, Daishu Han

**Affiliations:** 1 School of Basic Medicine, Peking Union Medical College, Institute of Basic Medical Sciences, Chinese Academy of Medical Sciences, Beijing, 100005, China; 2 Institute of Medical Biology, Chinese Academy of Medical Sciences, Kunming, 650118, China

## Abstract

Mumps virus (MuV) infection frequently causes orchitis and impairs male fertility. However, the mechanisms underlying the innate immune responses to MuV infection in the testis have yet to be investigated. This study showed that MuV induced innate immune responses in mouse Sertoli and Leydig cells through TLR2 and retinoic acid-inducible gene I (RIG-I) signaling, which result in the production of proinflammatory cytokines and chemokines, including TNF-α, IL-6, MCP-1, CXCL10, and type 1 interferons (IFN-α and IFN-β). By contrast, MuV did not induce the cytokine production in male germ cells. In response to MuV infection, Sertoli cells produced higher levels of proinflammatory cytokines and chemokines but lower levels of type 1 IFNs than Leydig cells did. The MuV-induced cytokine production by Sertoli and Leydig cells was significantly reduced by the knockout of TLR2 or the knockdown of RIG-I signaling. The local injection of MuV into the testis triggered the testicular innate immune responses *in vivo*. Moreover, MuV infection suppressed testosterone synthesis by Leydig cells. This is the first study examining the innate immune responses to MuV infection in testicular cells. The results provide novel insights into the mechanisms underlying the MuV-induced innate immune responses in the testis.

The testis is an immunoprivileged tissue wherein the systemic immune responses to autoantigens and alloantigens are remarkably reduced^1^. However, a broad spectrum of microbial pathogens, including viruses, bacteria, and parasites, can infect the testis via the circulating blood and the ascending genitourinary tract^2^. The innate immune responses in testicular cells overcome the immunoprivileged status and provide effective local innate defense against microbial infections^3^. The immune homeostasis in the testis is essential for normal spermatogenesis. The disruption of testicular immune homeostasis may lead to orchitis, an etiological factor of male subfertility and infertility^4^. Various viral infections can cause orchitis and perturb male fertility^5^,^6^. For instance, mumps virus (MuV) frequently causes orchitis and possibly leads to male infertility^5^. The underlying mechanisms of the innate immune responses to MuV infection in the testis and their effect on testicular function have yet to be intensively investigated.

Pattern recognition receptors (PRRs) rapidly initiate the innate immune responses upon recognition of conserved molecular patterns of microorganisms^7^. Three main families of PRRs, including Toll-like receptors (TLRs), retinoic acid-inducible gene I (RIG-I)-like receptors (RLRs), and cytosolic DNA sensors, have been well explored^8^. Viral infection triggers the innate immune responses through the activation of some TLRs, RLRs, and cytosolic DNA sensors^9^. TLRs are transmembrane proteins that can recognize viral proteins and nucleic acids. By contrast, two RLRs, namely, RIG-I and melanoma differentiation-associated protein 5 (MDA5), and cytosolic DNA sensors are located in the cytoplasm to recognize viral nucleic acids^10^. PRR activation initiates innate immune responses through various signaling pathways. TLR activation triggers myeloid differentiation protein 88-dependent and/or Toll/IL-1 receptor domain-containing adaptor inducing IFN-β-dependent pathways. RLRs initiate signaling pathway through adaptor IFN-β promoter stimulator-1 (IPS-1), whereas cytosolic DNA sensors use the stimulator of IFN gene^11^. These PRR signaling pathways induce the secretion of numerous proinflammatory cytokines, chemokines and type 1 interferons (IFN-α and IFN-β) through the activation of nuclear factor κB (NF-κB) and IFN-regulatory factors (IRFs), such as IRF3. These cytokines can regulate immune responses against microbial infections or directly restrict pathogen replication.

Some PRRs and their associated signaling adaptors are expressed in testicular cells. Mouse Sertoli cells express TLR2−TLR6^12^,^13^,^14^. Leydig cells express TLR2, TLR3, TLR4, RIG-I, MDA5 and cytosolic DNA sensor p204^15^,^16^,^17^. Male germ cells express TLR3 and TLR11^18^,^19^. PRR signaling in the testicular cells can be triggered by purified or synthetic ligands. However, PRR-initiated innate immune responses in the testicular cells to microbial infections remain unclear. In particularly, the MuV-triggered testicular innate immune responses should be investigated because MuV infection is highly testis tropic and severely impairs male fertility.

MuV is a negative-sense RNA virus that can cause mumps^20^. MuV infection also frequently causes mumps orchitis^21^. Mumps orchitis is associated with a direct viral infection in the testis^22^. Understanding the mechanisms of the innate immune responses to MuV infection in the testis can help develop preventive and therapeutic approaches to mumps orchitis. This study aimed to elucidate the PRR-initiated innate immune responses to MuV infection in mouse testicular cells.

## Results

### MuV-induced cytokine expression in testicular cells

**Figure 1:**
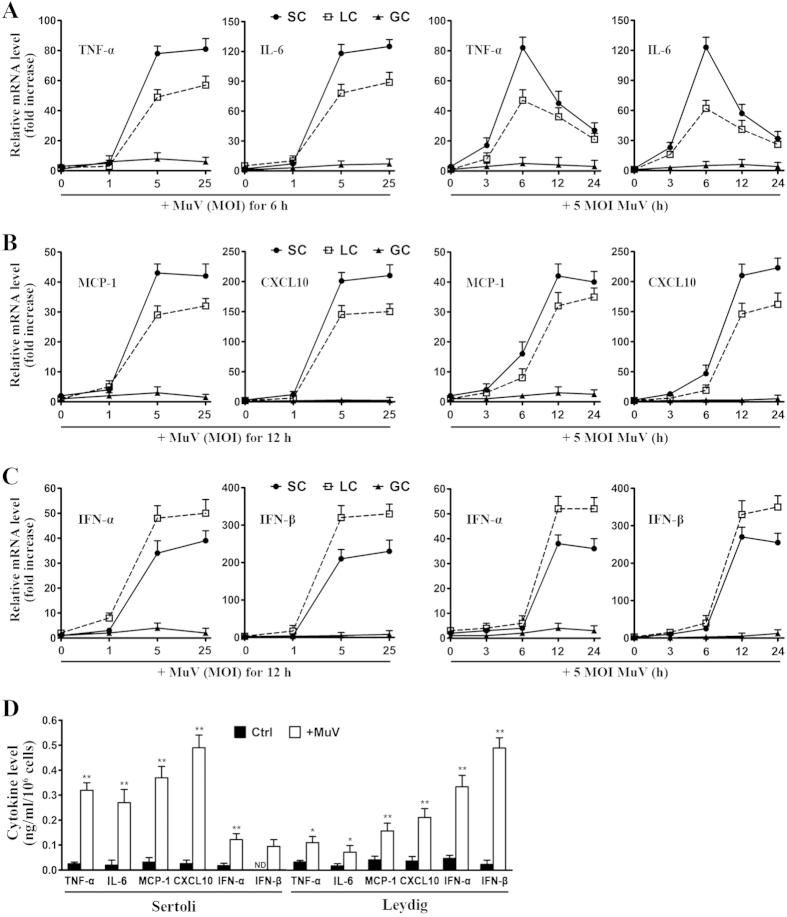
MuV-induced cytokine expression. Sertoli cells (SC), Leydig cells (LC), and male germ cells (GC) were isolated from C57BL/6 mice. (**A**) MuV-induced TNF-α and IL-6 expression. Cells were infected with different doses (multiplicity of infection, MOI) of MuV for 6 h (left panels) or with 5 MOI MuV for the specific durations (right panels). Total RNA was extracted from cells, and relative mRNA levels of TNF-α and IL-6 were determined with real-time qRT-PCR by normalizing to β-actin. The basal mRNA level in cells without MuV infection (point 0) was set as “1”. After the cells were infected with MuV, relative mRNA levels (fold increase) were determined after normalization to 1. (**B**) MCP-1 and CXCL10 expression. Cells were infected with the indicated MuV doses for 12 h (left panel) or with 5 MOI MuV for specific durations (right panel). The relative mRNA levels of MCP-1 and CXCL10 were determined with real-time qRT-PCR as described in (**A**). (**C**) Type 1 IFN expression. Cell were treated as described in (**B**). The relative mRNA levels of IFN-α and IFN-β in dose- (left panel) and time-dependent (right panels) manners were determined with real-time qRT-PCR. (**D**) Cytokine secretion. Sertoli and Leydig cells were infected wih 5 MOI MuV. After 24 h, the cytokine levels in the culture medium were measured using ELISA. The cells without MuV infection served as the controls (Ctrl). Data are presented as the means ± SEM of three independent experiments. *p < 0.05, **p < 0.01.

To examine the innate immune responses of testicular cells to MuV infection, we analyzed the expression of proinflammatory cytokines, chemokines, and type 1 IFNs in major testicular cell types, including Sertoli, Leydig, and germ cells. Real-time qRT-PCR results (Fig. 1A) showed that MuV infection remarkably increased the mRNA levels of TNF-α and IL-6 in a dose- (left panels) and a time-dependent manner (right panels) in Sertoli and Leydig cells. The mRNA levels of TNF-α and IL-6 peaked 6 h after the cells were infected with 5 MOI MuV. MuV infection also remarkably induced MCP-1 and CXCL10 expression in Sertoli and Leydig cells (Fig. 1B). The plateaus of the MCP-1 and CXCL10 mRNA levels were observed 12 and 24 h after the cells were infected with MuV. Moreover, MuV infection remarkably induced the mRNA levels of IFN-α and IFN-β in Sertoli and Leydig cells (Fig. 1C). By contrast, the MuV infection did not evidently induce the cytokine expression in male germ cells. ELISA results confirmed that MuV significantly induced the cytokine secretion by Sertoli and Leydig cells at 24 h after infection (Fig. 1D). Notably, Sertoli cells produced relatively high levels of TNF-α, IL-6, MCP-1, and CXCL10 but low levels of IFN-α and IFN-β compared with Leydig cells after MuV infection. MuV did not induce the male germ cells to secrete cytokines (data not shown).

### NF-κB and IRF3 activation

**Figure 2:**
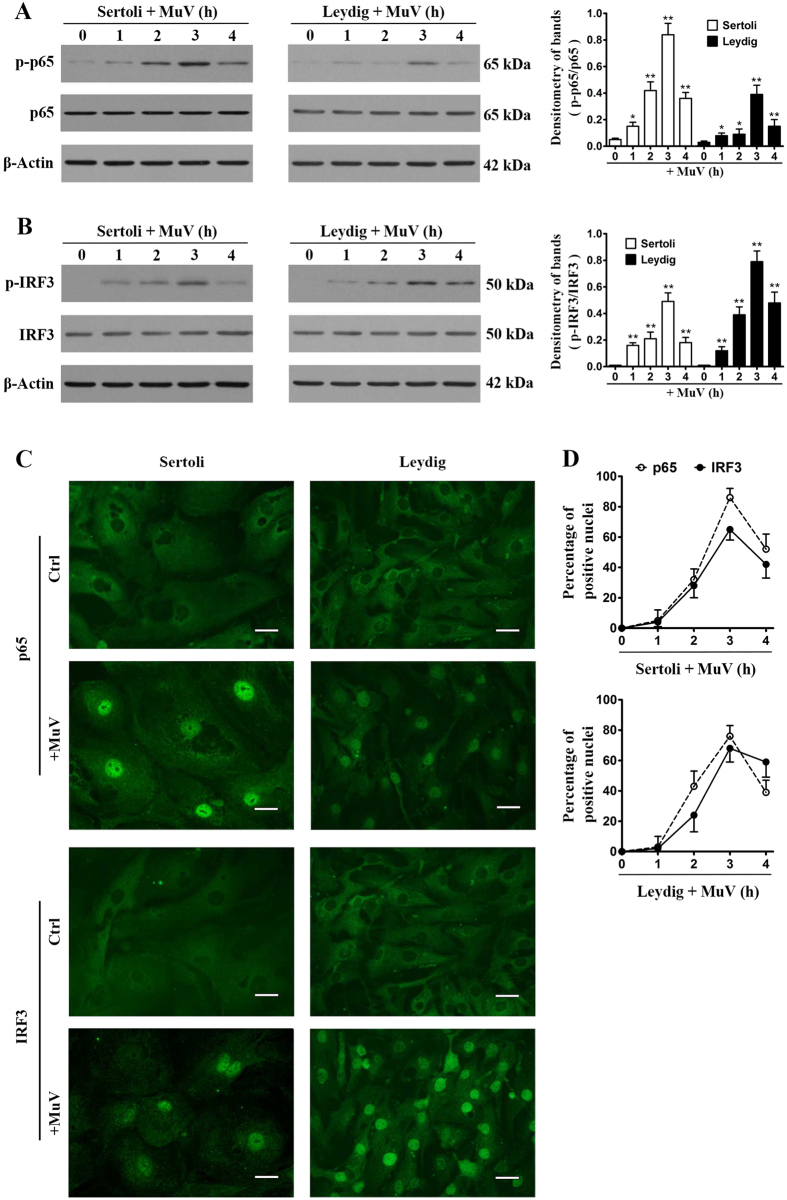
Activation of nuclear factor κB (NF-κB) and interferon regulatory factor 3 (IRF3). (**A**) NF-κB activation. Sertoli and Leydig cells were infected with 5 MOI MuV for the indicated hours (h). Cell lysates were subjected to Western blot to probe for phosphorylated NF-κBp65 (p-p65) and total p65 using specific antibodies. β-Actin was used as the loading control. Densitometry of bands (right panel) was analyzed with ImageJ software (http://rsb.info.nih.gov/ij). (**B**) IRF3 activation. Cells were treated as described in (**A**). Phosphorylated IRF3 (p-IRF3) and total IRF3 were determined by Western blot analysis. (**C**) Nuclear translocation of p65 and IRF3. Sertoli and Leydig cells were infected with 5 MOI MuV. The intracellular distribution of p65 (upper panels) and IRF3 (lower panels) was determined by indirect immunofluorescence staining. Images represent the nuclear translocation of p65 and IRF3 at 3 h after MuV infection. The uninfected cells served as the controls (Ctrl). Scale bar, 20 μm. (**D**) Dynamics of nuclear translocation. Cells were infected with MuV for specific durations. Nuclear translocation efficiencies of p65 and IRF3 were determined by spontaneously counting 100 cells for each experiment. Images represent at least three independent experiments. Data are presented as the means ± SEM of three experiments. *p < 0.05, **p < 0.01.

Given that NF-κB and IRF3 activation is necessary to induce cytokine expression^11^, we examined the phosphorylation of these transcription factors in Sertoli and Leydig cells after MuV infection. MuV evidently induced the phosphorylation of NF-κBp65 (p-p65) in Sertoli and Leydig cells in a time-dependent manner (Fig. 2A). The p-p65 level peaked at 3 h post MuV infection. Similarly, MuV infection also evidently induced the phosphorylation of IRF3 (p-IRF3) in both Sertoli and Leydig cells (Fig. 2B). The p-p65 and p-IRF3 must be translocated in the nucleus to induce cytokine expression. Indirect immunofluorescence staining confirmed the efficient nuclear translocation of p65 (Fig. 2C, upper panels) and IRF3 (lower panels) in Sertoli and Leydig cells at 3 h after MuV infection. By contrast, the nuclear translocation of p65 and IRF3 was not observed in the control cells that were not infected with MuV. A time-dependent p65 and IRF3 nuclear translocation efficiencies were analyzed by counting the Sertoli cells (Fig. 2D, upper panel) and Leydig cells (lower panel) whose nuclei are positive to p65 and IRF3.

### Involvement of NF-κB and IRF3 in MuV-induced cytokine expression

**Figure 3:**
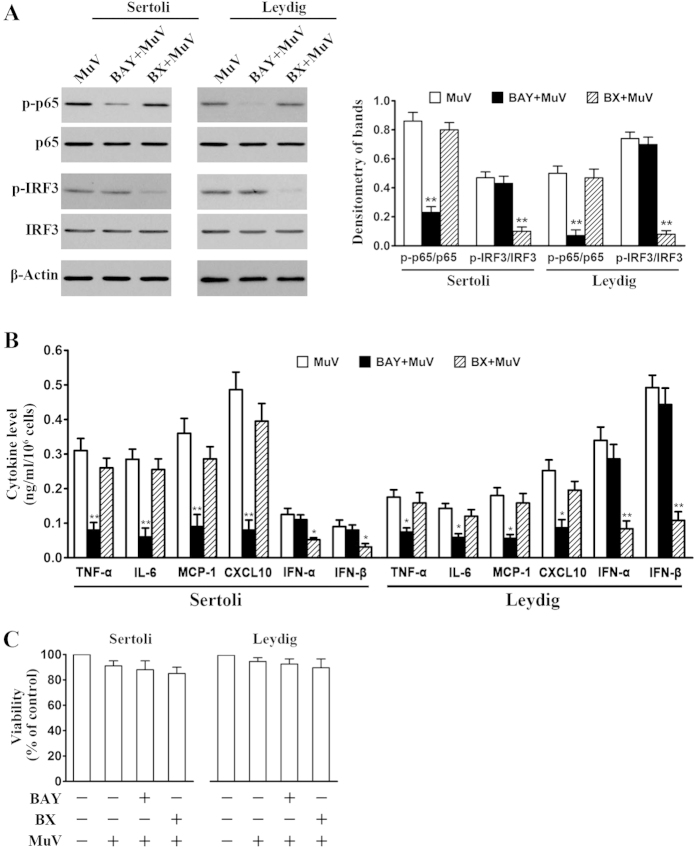
Involvement of NF-κB and IRF3 activation in cytokine expression. (**A**) Inhibition of NF-κB and IRF3 activation. Sertoli and Leydig cells were infected with 5 MOI MuV alone, or with MuV after a 2-h pretreatment with 10 μM BAY11-7082 (BAY), or with MuV after a 2-h pretreatment with 1 μg/ml BX795 (BX). At 3 h post MuV infection, p-p65 and p-IRF3 levels were determined by Western blot (left panels). Densitometry of bands was analyzed with ImageJ software (right panel). (**B**) Effects of BAY11-7082 and BX795 on cytokine secretion. Sertoli and Leydig cells were treated as described in (**A**). The cytokine levels in the culture medium were measured with ELISA at 24 h post MuV infection. (**C**) Cell viability. Cells were treated as described in (**A**). Cell viability was assessed using an MTT assay at 24 h post MuV infection. Images represent at least three independent experiments. Data are presented as the means ± SEM of three experiments. *p < 0.05, **p < 0.01.

We examined the effects of the chemical inhibitors of NF-κB and IRF3 activation on the MuV-induced cytokine expression to confirm the involvement of NF-κB and IRF3 in the innate immune responses. MuV-induced p-p65 and p-IRF3 levels in Sertoli cells were evidently reduced by a 2-h pretreatment of cells with BAY11-7082 (inhibitor of IκBα degradation) and BX795 (inhibitor of TBK1), respectively (Fig. 3A, left panels). The similar effects of the inhibitors on p65 and IRF3 phosphorylation were observed in Leydig cells (Fig. 3A, middle panels). BAY11-7082 and BX795 significantly inhibited MuV-induced phosphorylation of p65 and IRF3, respectively, in both Sertoli and Leydig cells (Fig. 3A, right panel). Accordingly, BAY11-7082 significantly inhibited the secretion of TNF-α, IL-6, MCP-1 and CXCL10 by Sertoli and Leydig cells (Fig. 3B). BAY-7082 did not significantly affect IFN-α and IFN-β secretion. By contrast, BX795 significantly inhibited IFN-α and IFN-β secretion but did not significantly affect the production of TNF-α, IL-6, MCP-1 and CXCL10. MTT assay results showed that the inhibitor and MuV treatments did not significantly reduce cell viability (Fig. 3C).

### Role of TLR2 in MuV-induced cytokine secretion

**Figure 4:**
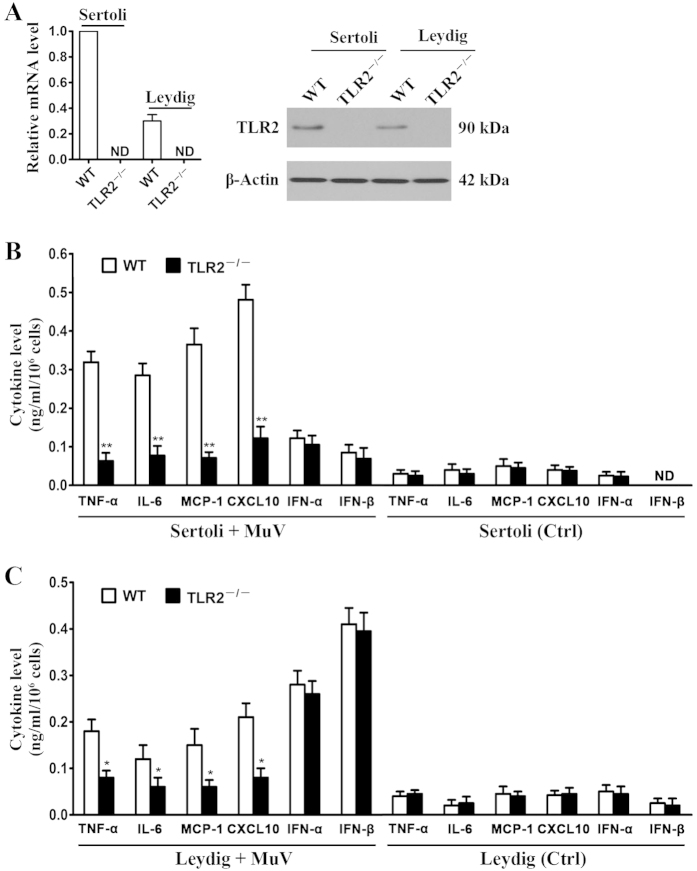
Role of TLR2 in MuV-induced cytokine production. (**A**) TLR2 expression. Sertoli and Leydig cells were isolated from three-week-old WT and TLR2^−/−^ mice. TLR2 mRNA level (left panel) was determined with real-time qRT-PCR by normalizing to β-actin. TLR2 protein was determined with Western blot using specific antibodies (right panel). (**B**) Cytokine production by Sertoli cells. The Sertoli cells of WT and TLR2^−/−^ mice were infected with 5 MOI MuV for 24 h (left panel). The cells without MuV infection served as the controls (Ctrl). Cytokine levels in culture medium were measured with ELISA. (**C**) Cytokine production by Leydig cells. WT and TLR2^−/−^ Leydig cells were treated as described for Sertoli cells in (**B**). The cytokine levels in the culture medium were measured with ELISA. Data are presented as the means ± SEM of three independent experiments. *p < 0.05, **p < 0.01.

Given that TLR2−TLR5 are expressed in Sertoli and Leydig cells^16^,^17^, we compared the cytokine secretion by WT and TLR knockout cells to determine the role of TLRs in MuV-induced innate immune responses. The cytokine expression was initially examined in the MuV-infected WT and TLR2^−/−^ cells. The absence of TLR2 in TLR2^−/−^ Sertoli and Leydig cells was confirmed at mRNA (Fig. 4A, left panel) and protein (right panel) levels. The mRNA and protein levels of TLR2 were relatively high in WT Sertoli cells compared with Leydig cells. ELISA results showed that TLR2^−/−^ Sertoli cells produced significantly low levels of TNF-α, IL-6, MCP-1, and CXCL10 compared with WT Sertoli cells at 24 h after MuV infection (Fig. 4B). By contrast, the levels of IFN-α and IFN-β produced by WT and TLR2^−/−^ Sertoli cells were comparable after MuV infection. In controls, the uninfected WT and TLR2^−/−^ Sertoli cells produced minimum cytokine levels (Fig. 4B, right panel). Likewise, TLR2^−/−^ Leydig cells produced significantly low levels of TNF-α, IL-6, MCP-1, and CXCL10 but yielded IFN-α and IFN-β levels comparable to those of WT Leydig cells after MuV infection (Fig. 4C). We also compared the cytokine expression in WT, TLR3^−/−^, TLR4^−/−^, and TLR5^−/−^ cells and found that MuV equally induced the cytokine secretion in WT and TLR knockout cells (data not shown). These results indicated that TLR2 signaling mediates proinflammatory cytokine and chemokine production but does not affect type 1 IFN production in Sertoli and Leydig cells in response to MuV infection.

### Involvement of RIG-I/IPS-1 signaling in MuV-induced cytokine secretion

**Figure 5:**
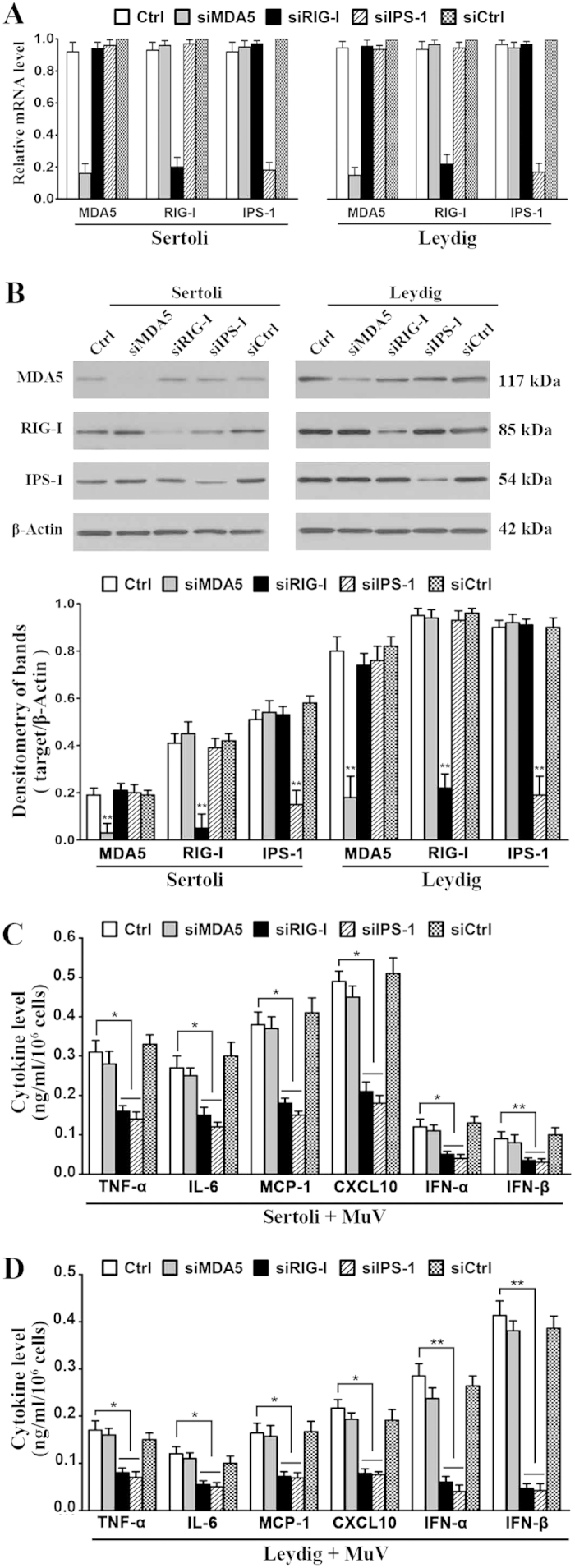
Roles of RIG-I/IPS-1 signaling in MuV-induced cytokine production. (**A**) Knockdown of RIG-I, MDA5 and IPS-1. Sertoli and Leydig cells were transfected with 100 nM siRNA targeting MDA5 (siMDA5), RIG-I (siRIG-I), IPS-1 (siIPS-1), or the control siRNA (siCtrl) targeting a scrambled sequence. After 24 h, the relative mRNA levels of the target genes were determined by real-time qRT-PCR. (**B**) Protein levels. Cells were transfected with siRNAs as described in (**A**). The protein levels of RIG-I, MDA5 and IPS-1 were determined by Western blot using specific antibodies at 24 h after siRNA transfection (upper panels). Densitometry of bands was analyzed with ImageJ software (lower panel). (**C**,**D**) Cytokine production. Sertoli cells (**C**) and Leydig cells (**D**) were transfected with siRNA as described in (**A**). After 24 h, cells were infected with 5 MOI MuV. The cytokine levels in the culture medium were measured using ELISA at 24 h after MuV infection. Images represent at least three independent experiments. Data are presented as the means ± SEM of three experiments. *p < 0.05, **p < 0.01.

Considering that mouse Sertoli and Leydig cells also express MDA5 and RIG-I^16^, we then examined the roles of MDA5, RIG-I, and their signaling adaptor IPS-1 in MuV-induced innate immune responses by using RNA interference approach. The cells were transfected with siRNA targeting MDA5 (siMDA5), RIG-I (siRIG-I), and IPS-1 (siIPS-1) for 24 h. Real-time qRT-PCR results showed that each siRNA specifically reduced the mRNA levels of respective target genes by >75% in Sertoli cells (Fig. 5A, left panel) and Leydig cells (right panel). A control siRNA (siCtrl) targeting a scrambled sequence did not affect the expression of MDA5, RIG-I and IPS-1. Western blot analysis confirmed that the protein levels of the target genes were significantly decreased in the cells after transfection with siRNA (Fig. 5B). The cells were then infected with 5 MOI MuV. The transfection of Sertoli cells with siRIG-I or siIPS-1 significantly reduced the cytokine levels at 24 h after MuV infection (Fig. 5C). Likewise, siRIG-I and siIPS-1 significantly inhibited the MuV-induced cytokine secretion by Leydig cells (Fig. 5D). By contrast, siMDA5 and siCtrl did not affect the cytokine production. MTT assay results showed that siRNA and MuV treatments did not significantly affect cell viability (see Supplementary Fig. S1 online). These results indicated that MuV also induced the innate immune responses in Sertoli and Leydig cells through RIG-I/IPS-1 signaling.

### MuV-induced testicular innate immune responses *in vivo*

**Figure 6:**
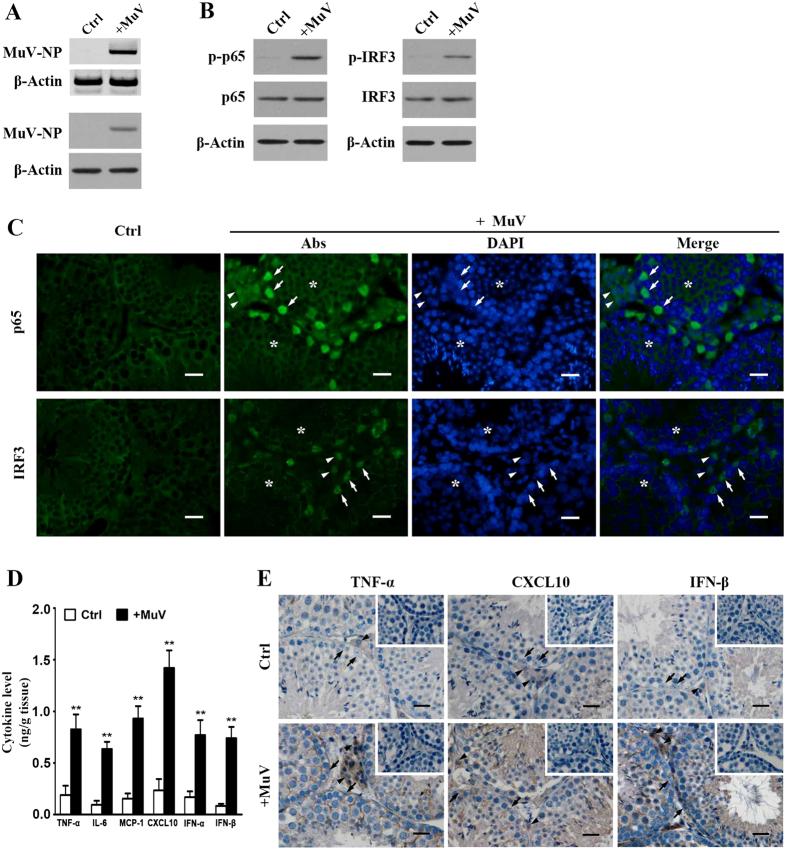
MuV-induced testicular innate immune responses *in vivo*. MuV (1 × 10^7^ plaque forming unit, PFU) in 10 μl of PBS was injected into one testis of five-week-old C57BL/6 mice. An equal volume of PBS alone was injected into the contralateral testis for the control (Ctrl). (**A**) Determination of MuV in the testis. Total RNA was extracted from the testis at 4 h after MuV infection. MuV nucleoprotein (MuV-NP) RNA was determined with RT-PCR (upper panels). MuV-NP protein was determined by Western blot (lower panels). β-Actin was used as control in RT-PCR and Western blot. (**B**) NF-κB and IRF3 activation. At 4 h post injection, p-p65 and total p65 (left panels), as well as p-IRF3 and total IRF3 (right panels) levels in the testicular lysates were determined with Western blot. (**C**) Nuclear translocation of p65 and IRF3. At 4 h post injection, the frozen sections of the testes were subjected to immunofluorescence staining to locate p65 (upper panels) and IRF3 (lower panels) using specific antibodies (Abs). The sections were counterstained with 4′, 6-diamidino-2-phenylindole (DAPI) to identify nuclei. (**D**) Cytokine levels. The testis was lysed by grinding in PBS at 24 h after injection. The cytokine levels in the lysates were measured with ELISA. (**E**) Cellular distribution of cytokines. The localization of TNF-α, CXCL10 and IFN-β was determined with immunohistochemical staining on the paraffin sections. Images represent at least three independent experiments. Scale bars, 20 μm. Arrows, arrowheads, and asterisks indicate Sertoli, interstitial, and germ cells, respectively. Data are presented as the means ± SEM of three experiments (n = 2 mice each experiment). **p < 0.01.

The testis of five-week-old C57BL/6 mice was locally injected with MuV to examine the testicular innate immune responses to MuV infection *in vivo*. MuV-NP mRNA (Fig. 6A, upper panel) and protein (lower panel) of MuV-NP in the testis were detected by RT-PCR and Western blot at 3 h after injection. MuV injection evidently induced the phosphorylation of p65 and IRF3 at 4 h (Fig. 6B). The p65 and IRF3 were efficiently translocated into the nuclei of Sertoli cells (arrows) and interstitial cells (arrowheads) at 4 h after MuV injection (Fig. 6C). Double immunofluorescence staining with anti-3β-HSD antibodies confirmed that p65 and IRF3 were translocated into the nuclei of Leydig cells (see Supplementary Fig. S2 online). By contrast, the nuclear translocation of p65 and IRF3 in Sertoli and interstitial cells was not observed in the control testis (Fig. 6C, left panels). We did not detect the nuclear translocation in germ cells after the testis was injected with MuV (asterisks). The cytokine levels in the testicular lysates were significantly increased at 24 h after MuV injection (Fig. 6D). Immunohistochemical staining showed that TNF-α, CXCL10 and IFN-β were predominantly localized in Sertoli cells (arrows) and interstitial cells (arrowheads) at 24 h after MuV injection (Fig. 6E, lower panels). The cytokine signals were not evident in the control testis (Fig. 6E, upper panels). To examine macrophages and germ cell apoptosis in the testis, we performed immunohistochemical staining for F4/80 and TUNEL staining. We did not find increased macrophages and apoptotic germ cells in the testis at 24 h post MuV infection compared to the control testis (see Supplementary Fig. S3 online).

### Inhibition of testosterone synthesis by MuV infection

**Figure 7:**
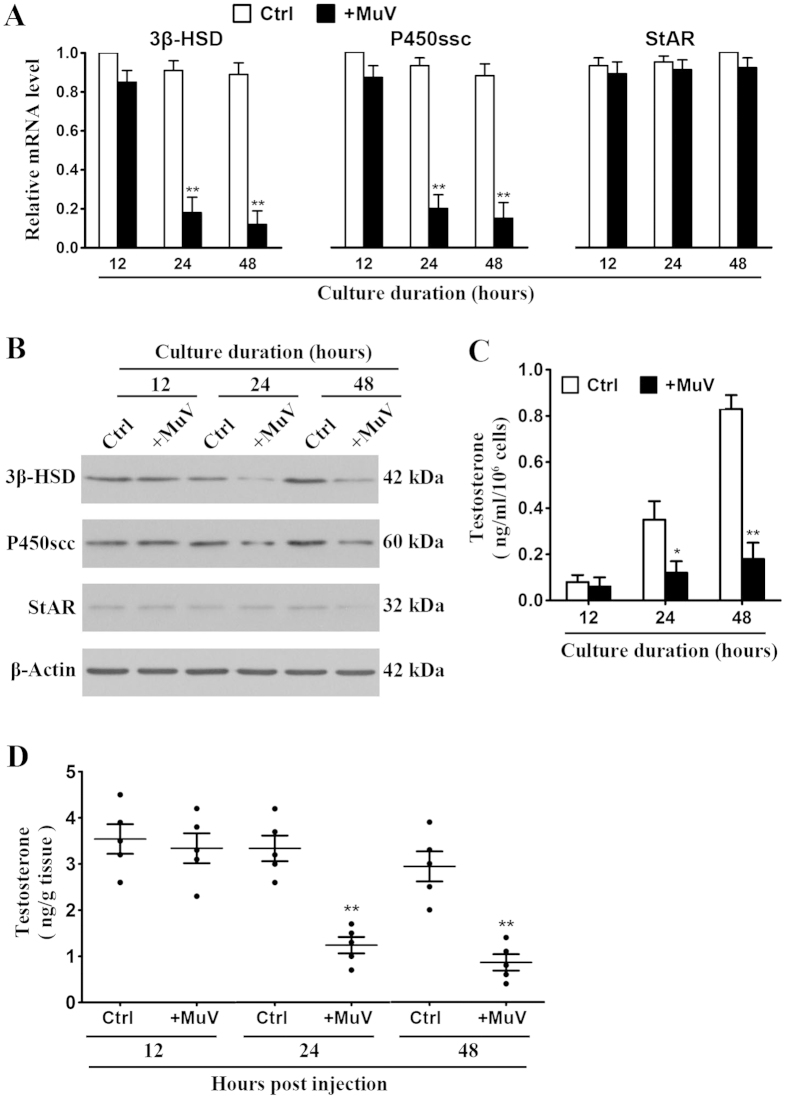
Effects of MuV on testosterone synthesis. (**A**) Expression of steroidogenic enzymes. Leydig cells of C57BL/6 mice were infected with 5 MOI MuV for specific durations. The uninfected cells served as controls (Ctrl). Total RNAs were extracted and the relative mRNA levels of 3β-hydroxysteroid dehydrogenase (3β-HSD), cytochrome P450 side-chain cleavage enzyme (P450scc), and steroidogenic acute regulatory protein (StAR) were determined with real-time qRT-PCR by normalizing to β-actin. (**B**) Protein levels of the enzymes. Leydig cells were infected as described in (**A**). The protein levels of 3β-HSD, P450scc and StAR were determined with Western blot using specific antibodies. (**C**) Testosterone level. Leydig cells were treated as described in (**A**). The testosterone level in the culture medium was measured with ELISA. (**D**) Testosterone synthesis *in vivo*. One testis of five-week-old C57BL/6 mice were injected with 1 × 10^7^ PFU MuV in 10 μl of PBS. The contralateral testis was injected with an equal volume of PBS alone for the Ctrl. At the indicated time points post injection, the testes were lysed and the testosterone level were measured with ELISA. Each dot indicates the testosterone level of individual testes. Data are means ± SEM (n = 5 mice). **p < 0.01.

Considering that MuV suppresses testosterone synthesis in human Leydig cells^23^, we examined the effect of MuV infection on the testosterone synthesis in mouse Leydig cells. Expression of the key steroidogenic enzymes 3β-hydroxysteroid dehydrogenase (3β-HSD) and cytochrome P450 side chain cleavage enzyme (P450scc) in primary Leydig cells was determined. Real-time qRT-PCR results showed that MuV infection inhibited the mRNA levels of 3β-HSD and P450scc in a time-dependent manner (Fig. 7A, left and middle panels). The significant decrease in 3β-HSD and P450scc mRNA levels was observed 24 and 48 h after the cells were infected with MuV. By contrast, MuV infection did not affect the expression of steroidogenic acute regulatory factor (StAR) in Leydig cells (Fig. 7A, right panel). The protein levels of 3β-HSD and P450scc were evidently reduced at 24 and 48 h after MuV infection (Fig. 7B). The protein level of StAR remained consistent in the control and MuV-infected cells. ELISA results showed that MuV significantly reduced the testosterone levels in culture media of Leydig cells at 24 and 48 h post infection (Fig. 7C). Furthermore, we analyzed the effect of MuV infection on testosterone synthesis *in vivo*. The local injection of MuV significantly reduced testosterone level in the testis at 24 and 48 h (Fig. 7D).

## Discussion

Although the incidences of mumps orchitis significantly declined due to the introduction of vaccination program in childhood, a global resurgence of the disease has been recently occurred^24^,^25^. Detection of MuV in the testicular biopsy and semen of mumps orchitis patients suggests that a direct viral infection in the testis is associated with the disease^22^,^26^. To understand the mechanisms underlying mumps orchitis, we elucidated the PRR-initiated innate immune responses to MuV infection in mouse testicular cells.

This study aimed to elucidate the innate immune responses to MuV infection in tissue-specific cells, including Sertoli, Leydig, and male germ cells, because these cells represent the majority of the testicular cells and the innate immune responses of these cells are less understood than those of immune cells. Although a considerable number of testicular macrophages reside in the interstitial spaces of the testis, we did not examine these macrophages because their innate immune responses have been intensively investigated^27^. The rat testicular macrophages produce fewer type 1 IFNs than Leydig cells after infection with Sendai viruses^28^. By contrast, the testicular macrophages predominantly produce anti-inflammatory cytokines and display immunosuppressive activities after the cells are activated^29^. We found that MuV significantly induced the innate immune responses in Sertoli and Leydig cells but not in male germ cells.

We examined the roles of PRRs in the MuV-induced innate immune responses in Sertoli and Leydig cells to reveal the underlying mechanisms. We found that MuV induced innate immune responses through TLR2 and RIG-I signaling. Several virus types, including respiratory syncytia virus, herpes simplex virus type 1, and lymphocytic choriomeningitis virus, induce innate immune responses via TLR2 signaling^30^,^31^,^32^. Our present study is the first work to reveal that TLR2 initiates the innate immune responses to MuV infection in testicular cells. However, the TLR2 ligand in viruses has yet to be identified. We found that MuV induced the production of proinflammatory cytokines and chemokines but not the production of type 1 IFNs in Sertoli and Leydig cells via TLR2. This result is agreement with previous studies demonstrating that TLR2 signaling may induce the production of different cytokines in terms of virus and cell types^33^. Although Sertoli and Leydig cells also express TLR3−TLR5, we excluded the involvement of these TLRs in the MuV-induced innate immune responses by using gene knockout cells (data not shown). The results indicated that TLR2 plays a crucial role in initiating the innate immune responses to MuV infection in testicular cells.

MuV also induced the innate immune responses in Sertoli and Leydig cells via RIG-I/IPS-1 signaling, which resulted in the production of proinflammatory cytokines, chemokines, and type 1 IFNs that we detected. Although MDA5 also initiates IPS-1-dependent signaling after activation by viral dsRNA, the knockdown of MDA5 with specific siRNA in Sertoli and Leydig cells did not significantly affect the cytokine production. This result suggests that MDA5 does not play a role in MuV-triggered innate immune responses. This result can be explained by the phenomenon that RIG-I and MDA5 recognize different structural patterns^34^. RIG-I senses short blunt-end dsRNA containing a 5′-triphoshate motif, whereas MDA5 recognizes long forms of viral dsRNA that is predominantly produced by positive-sense RNA viruses^35^,^36^. RIG-I agonists of MuV remain to be identified.

Type 1 IFNs are critical cytokines involved in the control of viral infection^37^. IFN-α and IFN-β induce the expression of numerous antiviral proteins that can restrict viral replication and degrade viruses^38^. IFNs can also activate the adaptive immune system against viral infection^39^. MuV significantly induced the production of IFN-α and IFN-β in Sertoli and Leydig cells via RIG-I/IPS-1 signaling but not via TLR2 signaling. Notably, Leydig cells produced relatively high level of IFN-α and IFN-β compared with Sertoli cells after MuV infection, suggesting that mouse Leydig cells have stronger antiviral capacities than Sertoli cells. However, a previous study showed that MuV induced weak antiviral responses in human Leydig cells^40^. These results suggest that mouse Leydig cells exhibit stronger antiviral responses than their human counterparts. In accordance with these observations, natural virus orchitis is not observed in mice while this this condition frequently occurs in humans. Therefore, understanding the underlying mechanisms of MuV-induced antiviral responses in the mouse and human testis should provide novel strategies for the development of preventive and therapeutic approaches to mumps orchitis. In this context, the innate immune responses to MuV infection in mouse and human testicular cells need to be comprehensively compared.

MuV infection also significantly induced the production of major pro-inflammatory cytokines and chemokines, including TNF-α, IL-6, MCP-1, and CXCL10, in Sertoli and Leydig cells. These cytokines promote inflammation by recruiting and activating leukocytes, which favor host defense against invading microbes. On the other hand, the inflammatory milieu may result in damage to the host. The inflammation in the testis can disrupt the immune privilege, thus leading to chronic orchitis^4^. The high level of TNF-α induces germ cell apoptosis in experimental autoimmune orchitis^41^. Leukocyte migration and activation can increase proinflammatory cytokine levels, thereby enhancing the damage to the host self^42^. Accordingly, MCP-1 and CXCL10 were involved in the onset of orchitis^43^. Moreover, the high levels of TNF-α and IL-6 perturb steroidogenesis^44^. MuV infection inhibited testosterone synthesis in human Leydig cells^23^. Our present study confirmed that MuV infection inhibited steroidogenic enzyme expression in mouse Leydig cells and suppressed testosterone synthesis *in vitro* and *in vivo*. Notably, Sertoli cells secreted relatively high level of proinflammatory cytokines and chemokines compared with Leydig cells in response to MuV infection. This result suggests that Sertoli cells may predominantly contribute to inflammation in the testis after MuV infection. Nevertheless, the role of testicular cells in the onset of mumps orchitis should be future investigated.

In summary, this study showed that MuV induced innate immune responses in mouse Sertoli and Leydig cells through TLR2 and RIG-I/IPS-1 signaling. Furthermore, MuV infection inhibited testosterone synthesis in Leydig cells. These results suggest that Sertoli and Leydig cells should be involved in the testicular defense against MuV, which provide novel insights into the mechanisms underlying the testicular innate immune responses to MuV infection.

## Materials and Methods

### Animals

C57BL/6 mice were obtained from the Laboratory Animal Center of Peking Union Medical College (Beijing, China). TLR2 knockout (TLR2^−/−^, B6.129-Tlr2tm1Kir/J) mice with original genetic background of 50% C57BL/6 and 50% 129S1 were purchased from the Jackson Laboratory (Bar Harbor, Maine, USA). Wild-type (WT) mice were obtained by backcrossing TLR2^−/−^ mice to C57BL/6 strain. The mice were maintained in a specific pathogen-free facility with a 12 h/12 h light/dark cycle and were provided with food and water ad libitum. All experimental procedures were performed according to the Guidelines for the Care and Use of Laboratory Animals established by the Chinese Council, and were approved by Institutional Animal Care and Use Committee of Institute of Basic Medical Sciences in China.

### Major reagents and antibodies

The small interfering RNA (siRNA) targeting mouse MDA-5 (sc-61011), RIG-I (sc-51481), IPS-1 (sc-75756), and control siRNA (sc-37007) targeting a scrambled sequence were purchased from Santa Cruz Biotechnology. BAY11-7082 (tlrl-b82) and BX795 (tlrl-bx7) were purchased from InvivoGen (San Diego, CA, USA). *In Situ* Cell Death Detection Kit (POD 11684817910) was purchased from Roche Diagnostics GmbH (Mannheim, Germany). The antibodies used in this study are listed in Table 1.

### Cell isolation

Leydig and Sertoli cells were isolated from three-week-old mice, and male germ cells were isolated from five-week-old mice, based on previously described procedures^15^,^16^. In brief, the testes of three mice were decapsulated and incubated with 0.5 mg/ml collagenase type 1 (Sigma) in F12/DMEM (Life Technologies, Grand Island, NY, USA) at room temperature for 15 min with gentle oscillation. The suspensions were filtered through 80 μm copper mesh to separate interstitial cells from seminiferous tubules. The interstitial cells were cultured in F12/DMEM supplemented with 100 U/ml penicillin, 100 mg/ml streptomycin, and 10% fetal calf serum (FCS, Life Technologies). After 24 h, Leydig cells were detached by 0.125% trypsin treatment for 5 min. The testicular macrophages were not detached by this treatment. Leydig cell purity was >92% based on staining for 3β-hydroxysteroid dehydrogenase, a marker of Leydig cells^45^. The macrophages in Leydig cell preparations were <5% based on the immunostaining for F4/80, a marker of macrophages^46^. The other minor contaminant cells were presumably fibroblasts and vascular endothelial cells.

The seminiferous tubules were recovered and suspended in collagenase type I at room temperature for 15 min to remove peritubular myoid cells. The tubules were cut into small pieces of approximately 1 mm and incubated with 0.5 mg/ml hyaluronidase (Sigma) at room temperature for 10 min with gentle pipetting to dissociate germ cells from Sertoli cells. Suspensions were cultured with F12/DMEM at 32 °C for 6 h. The germ cells were recovered by collecting non-adherent cells. The germ cell purity was >95% based on cell nuclear morphology after staining with 4′, 6-diamidino-2-phenylindole^18^. Sertoli cells were cultured for additional 24 h and then treated with a hypotonic solution (20 mM Tris, pH 7.4) for 1 min to remove germ cells adhering to Sertoli cells. The purity of Sertoli cells was >95% based on the immunostaining for Wilms tumor nuclear protein 1, a marker of Sertoli cells^47^.

### MuV infection

MuV (SP-A strain) was isolated from a mumps patient^48^, and obtained from the Institute of Medical Biology, Chinese Academy of Medical Sciences (Kunming, China). MuV was amplified and titrated in Vero cells. MuV preparations were diluted in 1× PBS at a density of 1 × 10^9^ plaque forming unit (PFU)/ml and stored at −80 °C. MuV was added to cell cultures for infection *in vitro*. The infection efficiency of MuV was >90% in testicular cells, including Sertoli, Leydig, and germ cells. Five-week-old mice were anesthetized with pentobarbital sodium (50 mg/kg) and the testes were surgically exposed to infect the testis *in vivo*. One testis was locally injected with 1 × 10^7^ PFU MuV in 10 μl of PBS by using 30-gauge needles. The contralateral testis was injected with an equal volume of PBS for the control.

### 3-(4,5-dimethylthiazolyl-2)-2,5-diphenyl-tetrazolium bromide (MTT) assay

Cell viability was assessed using an MTT assay kit (ATCC, Manassas, VA, USA) in accordance with the manufacturer’s instructions. In brief, cells were cultured in 96-well microplates at a density of 2 × 10^4^ cells/well. After treatments with MuV and chemical inhibitors, the cells were incubated with 10 μl of MTT solution. After 2 h, 100 μl of the detergent reagent, which was included in kit, was added to each well. The absorbance at 570 nm was determined with a microplate reader (BioTek, Winooski, VT, USA). The percentage of the absorbance value versus the control value represents cell viability.

### Real-time qRT-PCR

Total RNA was extracted with Trizol^TM^ reagent (Invitrogen, Carlsbad, CA, USA) in accordance with the manufacturer’s instructions. The RNA was treated with RNase-free DNase 1 (Invitrogen) to remove potential DNA contaminants. The absence of the genomic DNA was confirmed by the PCR amplification of β-actin for 35 cycles before reverse transcription. RNA (1 μg) was reverse transcribed into cDNA in a 20 μl reaction mixture containing 2.5 μM random hexamers, 2 μM dNTPs, and 200 U Moloney murine leukemia virus reverse transcriptase (Promega, Madison, WI, USA). PCR was performed using a Power SYBR^®^ Green PCR master mix kit (Applied Biosystems, Fostercity, CA, USA) on an ABI PRISM 7300 real-time cycler (Applied Biosystems). The relative mRNA levels were determined by the comparative 2^−ΔΔCT^ method as described in the Applied Biosystems User Bulletin No. 2 (P/N 4303859)^49^. The primer sequences are listed in Table 2.

### Western blot analysis

The testes or cells were lysed with a lysis buffer (Applygen Technologies Inc., Beijing, China), and the protein concentrations were determined using a bicinchoninic acid protein assay kit (Pierce Biotechnology, Rockford, IL, USA). The proteins (20 μg/well) per well were separated on 10% SDS-PAGE gel and then electrotransferred onto PVD membranes (Millipore, Bedford, MA, USA). The membranes were blocked with Tris-buffered saline (TBS, pH 7.4) containing 5% non-fat milk at room temperature for 1 h and then incubated with the primary antibodies overnight at 4 °C. The membranes were washed twice with TBS containing 0.1% Tween-20 and then incubated with the horseradish-peroxidase (HRP)-conjugated secondary antibodies at room temperature for 1 h. Ag/Ab complexes were visualized using an enhanced chemiluminescence detection kit (Zhongshan Biotechnology Co.). β-Actin was used as the loading control. Band densitometry was quantified using ImageJ software (http://rsb.info.nih.gov/ij).

### Immunohistochemistry and immunofluorescence staining

The testes were fixed in Bouin’s solution for 24 h and then embedded in paraffin. The paraffin sections (5 μm thick) were prepared using rotary microtome Reichert 820 HistoSTAT (Reichert Technologies, NY, USA). In frozen sections, the testes were fixed in 4% paraformaldehyde for 24 h. After cryoprotection in 30% sucrose, the tissues were cut to 7 μm thick sections using Leica CM1950 apparatus (Leica Biosystems, Nussloch, Germany). The sections were incubated with 3% H_2_O_2_ in PBS for 15 min to inhibit endogenous peroxidase activity. The slides were soaked in citrate buffer and then heated in a microwave at 100 °C for 10 min to retrieve the antigens. After blocking with 5% normal goat sera in PBS for 1 h at room temperature, the sections were incubated with the primary antibodies overnight at 4 °C. After washing twice with PBS, the sections were incubated with the appropriate FITC- or HRP-conjugated secondary antibodies at room temperature for 30 min. HRP activity was visualized via the diaminobenzidine method. Negative controls were incubated with pre-immune rabbit sera instead of primary antibodies. The sections were counterstained with DAPI for immunofluorescence staining or with hematoxylin for immunohistochemical staining, and then mounted with neutral balsam (Zhongshan Biotechnology Co.).

For indirect immunofluorescence staining on cultured cells, the cells were fixed with pre-cooled methanol at −20 °C for 3 min and then permeabilized with 0.2% Triton X-100 in PBS for 15 min. The cells were blocked with 5% normal goat sera at room temperature for 30 min and then incubated with primary antibodies at 37 °C for 90 min. The cells were washed twice with PBS and incubated with appropriate FITC-conjugated secondary antibodies (Zhongshan Biotechnology Co.) for 30 min. The cells were mounted with neutral balsam and observed under a fluorescence microscope BX-51 (Olympus, Tokyo, Japan).

### ELISA

The culture media were collected at 24 h after the cells were infected by MuV. The testis was lysed by grinding in 1 × PBS, and the supernatants of the lysates were collected after centrifugation at 800 *g* for 5 min. The cytokine and testosterone levels were measured using ELISA kits in accordance with the manufacturer’s instructions. The mouse TNF-α (CME0004) and IL-6 (CME0006) ELISA kits were purchased from Beijing 4 A Biotech Company (Beijing, China). The mouse MCP-1 (KB3817A), CXCL10 (BMS6018) and IFN-α (BMS6027) ELISA kits were purchased from eBioscience (San Diego, CA, USA). The ELISA kits for IFN-β (42400) and for mouse testosterone (DEV 9911) were purchased from R&D Systems (Minneapolis, MN, USA) and Demedtec (Kiel-Wellsee, Germany), respectively.

### Statistical analysis

Data were presented as the mean ± SEM of at least triplicate experiments. Statistical difference between two groups was determined using Student’s *t*-test. One-way ANOVA with Bonferroni’s (selected pairs) post hoc test was used to compare more than two groups. The calculations were performed with SPSS version 13.0 (SPSS Inc., Chicago, IL, USA). *P* < 0.05 was considered statistically significant.

## Additional Information

**How to cite this article**: Wu, H. *et al*. Mumps virus-induced innate immune responses in mouse Sertoli and Leydig cells. *Sci. Rep.* **6**, 19507; doi: 10.1038/srep19507 (2016).

## Supplementary Material

Supplementary Information

## Figures and Tables

**Table 1:** Antibody used in this study.

Vendor	Antibody	Host species	Catalog number	Use	Working dilution	Conjugation
Sigma–Aldrich, St. Louis, MO, USA	anti-β-actin	Mouse	A5316	WB	1:4000	
Abcam, Cambridge, UK	anti-TNF-α	Rabbit	ab9739	IHC	1:200	
	anti-CXCL10	Rabbit	ab9938	IHC	1:200	
	anti-IFN-β	Rabbit	ab140211	IHC	1:200	
	anti-IPS-1	Rabbit	ab31334	WB	1:500	
	anti-MDA5	Rabbit	ab69983	WB	1:1000	
	anti-RIG-I	Rabbit	ab45428	WB	1:1000	
	anti-P450scc	Rabbit	ab175408	WB	1:1000	
	anti-StAR	Rabbit	ab96637	WB	1:1000	
	anti-MuV nucleoprotein (NP)	Mouse	ab9876	WB	1:500	
	anti-TLR2	Rat	ab109810	WB	1:1000	
	anti-F4/80	Rat	Ab6640	IF	1:200	
Cell Signaling, Beverly, MA	anti-NF-κBp65	Rabbit	4764	WB/IF	1:1000/1:200	
	anti-phospho-NF-κBp65	Rabbit	3031	WB	1:1000	
	anti-phospho-IRF3	Rabbit	3661	WB	1:1000	
Santa Cruz Biotechnology, Santa Cruz, CA, USA	anti-IRF3	Rabbit	sc-9082	WB/IF	1:1000/1:200	
	anti-3β-HSD	Goat	sc-30820	WB	1:1000	
Zhongshan Biotechnology Co., Beijing, China	Rabbit IgG	Goat	ZF-0311	IF	1:200	FITC
	Rabbit IgG	Goat	ZB-2301	WB/IHC	1:4000/1:200	HRP
	Mouse IgG	Goat	ZB-2305	WB	1:4000	HRP
	Goat IgG	Rabbit	ZB-2306	WB	1:4000	HRP
	Rat IgG	Goat	ZB-2307	WB	1:4000	HRP

WB, Western blot; IHC, immunohistochemistry; IF, immunofluorescence staining; FITC, fluorescein isothiocyanate; HRP, horseradish peroxidase.

**Table 2:** Primers used for real-time PCR.

Target genes	Primer pairs (5′ → 3′)	
	Forward	Reverse
IL-6	ATTCCTCTGTGCCACCTTTAC	GGTCAGCACCACCATCTTATT
TNF-α	CATCTTCTCAAAATTCGAGTGACAA	TGGGAGTAGACAAGGTACAACCC
CXCL-10	CCAAGTGCTGCCGTCATTTTC	TCCCTAAGGCCCTCATTCTCA
MCP-1	TTAAAAACCTGGATCGGAACCAA	GCATTAGCTTCAGATTTACGGGT
IFN-α	TTCCTCAGACTCATAACCTCAGGA	ATTTGTACCAGGAGTGTCAAGGC
IFN-β	GACGTGGGAGATGTCCTCAAC	GGTACCTTTGCACCCTCCAGTA
TLR2	GCAAACGCTGTTCTGCTCAG	AGGCGTCTCCCTCTATTGTATT
MDA5	AGATCAACACCTGTGGTAACACC	CTCTAGGGCCTCCACGAACA
RIG-I	CCACCTACATCCTCAGCTACATGA	TGGGCCCTTGTTGTTCTTCT
IPS-1	AGACACCAAGTTGCCCCAAG	CTGGAAGGAAACGGTTGGAGA
3β-HSD	TATTCTCGGTTGTACGGGCAA	GTGCTACCTGTCAGTGTGACC
P450scc	AGGTCCTTCAATGAGATCCCTT	TCCCTGTAAATGGGGCCATAC
StAR	ATGTTCCTCGTCACGTTCAAG	CCCAGTGCTCTCCAGTTGAG
MuV-NP	TCAGATCAATCGCATCGGGG	CTTGCGACTGTGCGTTTTGA
β-Actin	GAAATCGTGCGTGACATCAAAG	TGTAGTTTCATGGATGCCACAG
