# Supplementary material for: Mumps virus-induced innate immune responses in mouse Sertoli and Leydig cells
Source: Sci Rep. 2016 Jan 18;6:19507. doi: 10.1038/srep19507 (PMC4725973; doi:10.1038/srep19507)
Supplement: Supplementary Information [file srep19507-s1.pdf]

Mumps virus-induced innate immune responses in mouse Sertoli and Leydig cells

Han Wu[1], Lili Shi[1], Qing Wang[1], Lijing Cheng[1], Xiang Zhao[1], Qiaoyuan Chen[1], Qian Jiang[1], Min Feng[2], Qihan Li[2], Daishu Han[1,*]

[1]School of Basic Medicine, Peking Union Medical College, Institute of Basic Medical Sciences, Chinese Academy of Medical Sciences, Beijing, 100005, China.

[2]Institute of Medical Biology, Chinese Academy of Medical Sciences, Kunming, 650118, China.

*Correspondence and requests for materials should be addressed to D.H. (email: dshan@ibms.pumc.edu.cn.)

Legends to Supplementary Figures.

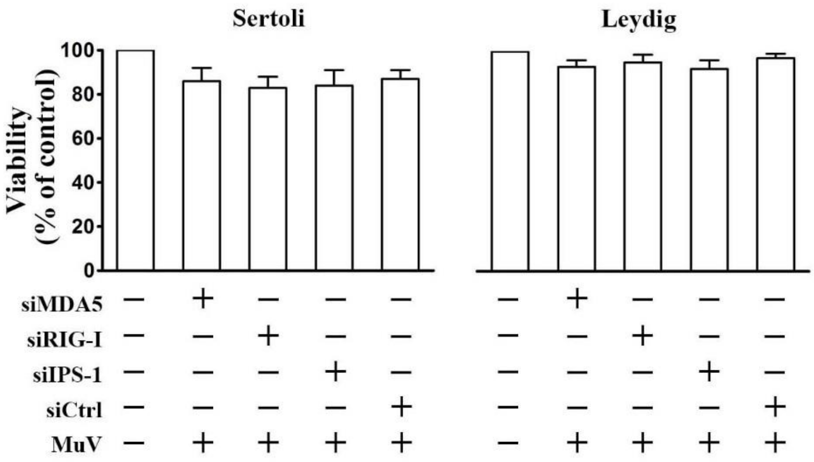

Supplementary Figure S1. Cell viability. Sertoli and Leydig cells were transfected with siRNA for 24 h, and followed by MuV infection. Cell viability was assessed using an MTT assay at 24 h post MuV infection. Data are presented as the means SEM of three experiments.

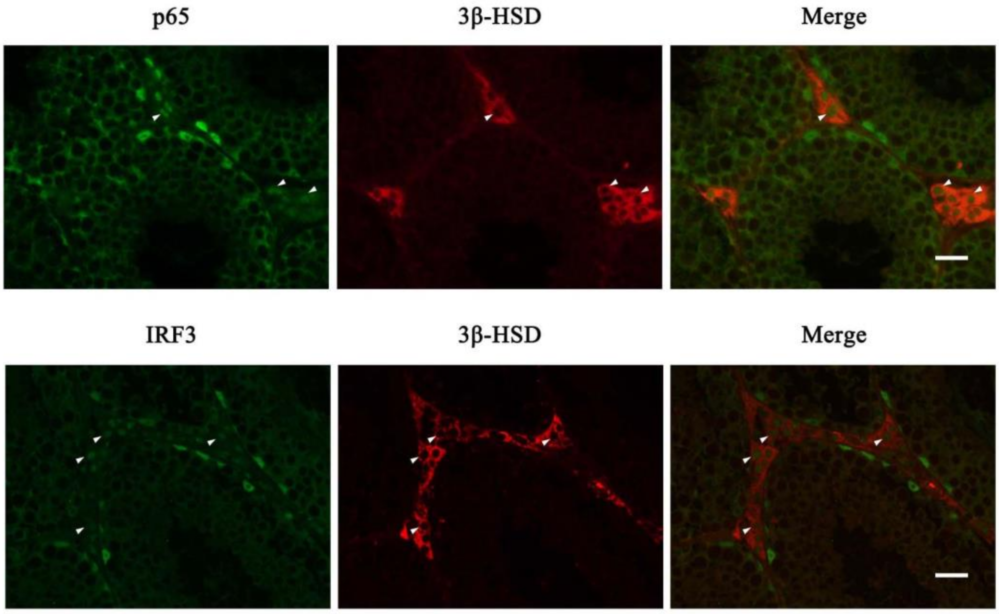

Supplementary Figure S2. Double immunofluorescence staining of p65 or IRF3 and 3β-HSD (Leydig cell marker). At 4 h post MuV injection, the frozen sections of the testis were subjected to double immunofluorescence staining to localize p65 or IRF3 (left panels) and 3β-HSD (middle panels) using specific antibodies. The co-localization of p65 or IRF3 and 3β-HSD are visible in merged images (right panels). Images represent three independent experiments. Arrowheads indicate Leydig cells. Scale bars, 20 μm.

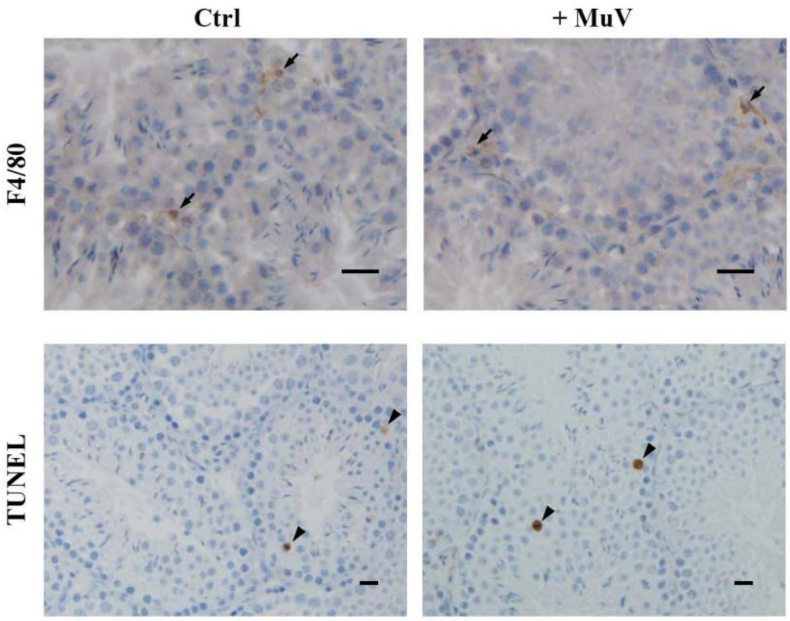

Supplementary Figure S3. Macrophages and apoptotic germ cells. One testis of five-week-old C57BL/6 mice were injected with 1 × 10^7 PFU MuV in 10 μl of PBS (right panel). The contralateral testis was injected with an equal volume of PBS alone for the Ctrl (left panels). At 24 h post MuV injection, paraffin sections of the testes were subjected to immunohistochemical staining for F4/80 (macrophage marker) using specific antibodies (upper panels) and TUNEL staining using In Situ Cell Death Detection Kit (lower panels). Images represent three independent experiments. Arrows and arrowheads indicate macrophages (upper panels) and apoptotic germ cells (lower panels), respectively. Scale bars, 20 μm.
